# Clinical anatomy and 3D virtual reconstruction of the lumbar plexus with respect to lumbar surgery

**DOI:** 10.1186/1471-2474-12-76

**Published:** 2011-04-14

**Authors:** Sheng Lu, Shan Chang, Yuan-zhi Zhang, Zi-hai Ding, Xin Ming Xu, Yong-qing Xu

**Affiliations:** 1 Department of Orthopedics, Kunming General Hospital, Chengdu Military Region, PLA, Kunming, China; 2 Department of Orthopedics, the First Hospital of Chengdu Medical College, Chengdu, China; 3 Department of Orthopedics, the First Hospital Affiliated to the Inner Mongolia Medical College, Hohhot, Inner Mongolia Autonomous Region, China; 4 The Clinical Anatomy Institute, the Nan Fang Medical University, Guangzhou, China

## Abstract

**Background:**

Exposure of the anterior or lateral lumbar via the retroperitoneal approach easily causes injuries to the lumbar plexus. Lumbar plexus injuries which occur during anterior or transpsoas lumbar spine exposure and placement of instruments have been reported. This study aims is to provide more anatomical data and surgical landmarks in operations concerning the lumbar plexus in order to prevent lumbar plexus injuries and to increase the possibility of safety in anterior approach lumbar surgery.

**Methods:**

To study the applied anatomy related to the lumbar plexus of fifteen formaldehyde-preserved cadavers, Five sets of Virtual Human (VH) data set were prepared and used in the study. Three-dimensional (3D) computerized reconstructions of the lumbar plexus and their adjacent structures were conducted from the VH female data set.

**Results:**

The order of lumbar nerves is regular. From the anterior view, lumbar plexus nerves are arranged from medial at L5 to lateral at L2. From the lateral view, lumbar nerves are arranged from ventral at L2 to dorsal at L5. The angle of each nerve root exiting outward to the corresponding intervertebral foramen increases from L1 to L5. The lumbar plexus nerves are observed to be in close contact with transverse processes (TP). All parts of the lumbar plexus were located by sectional anatomy in the dorsal third of the psoas muscle. Thus, access to the psoas major muscle at the ventral 2/3 region can safely prevent nerve injuries. 3D reconstruction of the lumbar plexus based on VCH data can clearly show the relationships between the lumbar plexus and the blood vessels, vertebral body, kidney, and psoas muscle.

**Conclusion:**

The psoas muscle can be considered as a surgical landmark since incision at the ventral 2/3 of the region can prevent lumbar plexus injuries for procedures requiring exposure of the lateral anterior of the lumbar. The transverse process can be considered as a landmark and reference in surgical operations by its relative position to the lumbar plexus. 3D reconstructions of the lumbar plexus based on VCH data provide a virtual morphological basis for anterior lumbar surgery.

## Background

Exposing the anterior or lateral lumbar using the retroperitoneal approach assumes one of two methods: manipulating the major vessels inward or incising the lateral psoas muscle (transpsoas approach). The latter procedure is the less risky of the two, but it could still cause injuries to the lumbar plexus [1-3]. Although the L5 nerve is not a part of the lumbar plexus, its intimate relationship with the lumbar plexus makes it interesting for this study. Lumbar plexus injuries, which can occur when exposing the spine and placing instruments therein, have been previously reported [4,5]. Extreme lateral interbody fusion (XLIF) is a relatively new technique whereby access to the disc space is achieved through a minimally invasive lateral, retroperitoneal, trans-psoas approach. The nerves of the lumbar plexus reside within the psoas [6,1]. The potential complications of the lateral approach are mostly related to the psoas and the nerves of the lumbar plexus that lie within it. The nerves of the lumbar plexus and the genitofemoral nerve are at risk as the psoas muscle is traversed. Real-time EMG monitoring during this critical stage of the procedure can reliably detect the proximity of neural structures and signal the surgeon to redirect. Still, postoperative groin or thigh dysesthesias may occur in some patients. In one recent series of patients with degenerative lumbar scoliosis, 3 of 12 patients experienced transient groin or thigh dysesthesias [7].

Although the possibility of injury to the lumbar plexus during lumbar vertebrae exposure via the anterior retroperitoneal approach has been emphasized, surgeons face a dearth of anatomical knowledge on the lumbar plexus during this procedure. Once such knowledge becomes widely known, surgeons could either avoid nerve injury or determine the injury's location. However, applied anatomy of the lumbar plexus with respect to anterior lumbar surgery has not yet been reported [8,9]. More anatomical data concerning the lumbar plexus in clinical applications are needed in order to prevent lumbar plexus injuries while increasing the safety of lumbar surgery.

## Methods

Fifteen formaldehyde-preserved cadavers (come from the Nan Fang Medical University, had been approved by ethical committee for the use of these) with integral lumbar plexus were collected; 9 were male and 6 were female. The mean age of the cadavers was 69 years with a range of 56 to 87 years. Samples of 30 sides in total were observed and studied. Clearing away all organs in front of the peritoneum to expose the posterior abdomen cavity, the psoas major muscle was left intact in its original location as much as possible. The lumbar plexus was then dissected and exposed. The anatomical relationship of the lumbar plexus nerve and the surgical landmark was studied. Measurements of the plexus were taken with a flexible surgical ruler.

### Sectional anatomy of the lumbar plexus

Five sets of Virtual Human (VH) data from the Anatomic Department of South Medical University were used in this study. Sectional photos displayed the lumbar plexus nerve. The sectional plane was divided into three average sections. The upper part was the superior margin of psoas major, and the lower part was the intersection of the psoas major and lumboram muscles. The relationship of the psoas major and the lumbar plexus nerve was then observed.

### 3D virtual reconstruction of the lumbar plexus nerve

The TIFF format of VH Female 1 was translated to JPG format using Photoshop CS software. A part from L1 to the trochanter of the femur was selected. A total of 1,491 pictures were studied. The 3D-virtual model of the lumbar plexus nerve was established using Amira 3.1 software (TGS company, France) to observe the 3D relationship of the lumbar plexus and the vessel, vertebral body, kidney, and major psoas muscles.

## Results

### Anatomic observation of the lumbar nerve roots

**Figure 1:**
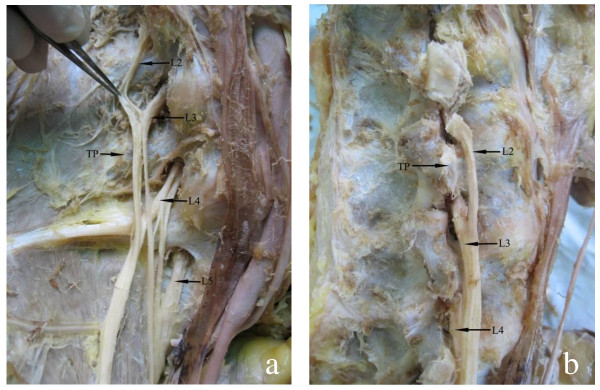
**Arrangement of the lumbar nerve, transverse process (TP)**. (A) anterior view, lumbar nerve, arranged from medial to lateral, from L5 to L2; (B) lateral view, lumbar nerve arranged from ventral to dorsal, from L2 to L5.

Lumbar nerve roots are situated in the posterior part of the psoas muscle. Majority travels across the corresponding inter-vertebral foramens, under the surface of the lumbar pedicle and across the transverse process (TP) ligament. The lumbar plexus is formed from tight loops, which widen in the lower vertebra. There are anatomic rules of the lumbar plexus in the lateral vertebra: lumbar nerves arrange from medial at L5 to lateral at L2 and from ventral at L2 to dorsal at L5 (Figure. 1).

### Angles of the lumbar plexus nerve to the intervertebral foramen

The angle of each root exiting outward to the corresponding intervertebral foramen ranges from 20° at L1 to 40° at L5; the largest angle is 32.9° at L5 (Table 1).

**Table 1:** Angle of each root exiting outward to the corresponding intervertebral foramen.

Nerve root	Angle to sagittal plane (degrees)
L1	20.4 ± 4.1
L2	22.1 ± 4.3
L3	26.9 ± 4.9
L4	30.4 ± 5.8
L5	32.9 ± 4.4

### Anatomic relationship of the lumbar plexus and the transverse process

The lumbar plexus is located anterolateral to the lumbar vertebrae. When it exits from the intervertebral foramina, it lays anterior to the transverse process for the duration. The transverse process was used as a landmark to protect the lumbar plexus during the operation. The distance between the upper level of the transverse process to its corresponding nerve trunk was 4.9-5.9 mm (3.6-7.9 mm). From L2 to L5, the distance between the lower level of the transverse process to its corresponding nerve trunk was 8.9 ± 1.0 mm (7.1-10.9 mm) at the level of L2, 7.8 ± 1.1 mm (5.3-9.4 mm) at L3, 6.8 ± 0.9 mm (4.9-8.4 mm) at L4, and 6.2 ± 0.9 mm (4.8-8.4 mm) at L5. Therefore, the distance between the inferomedial border of the transverse process and its corresponding nerve trunk decreases from the upper level to the lower level. Table 2 shows the relationship between the lumbar nerve and the transverse process.

**Table 2:** Relationship of lumbar nerve and transverse process (, mm)

Parameter measured	Distance(mm)			
	
	L2	L3	L4	L5
Superior margin of TP to nerve	4.9 ± 0.5 (3.6-5.3)	5.3 ± 0.8 (3.8-6.1)	5.9 ± 1.2 (3.6-7.9)	5.2 ± 0.7 (3.8-7.8)
Inferior margin of TP to nerve	8.9 ± 1.0 (7.1-10.9)	7.8 ± 1.1 (5.3-9.4)	6.8 ± 0.9 (4.9-8.4)	6.2 ± 0.9 (4.8-8.4)
Medial edge of vertebral to nerve on superior margin of TP	5.2 ± 1.2 (3.5-7.9)	5.0 ± 1.2 (3.1-7.6)	5.6 ± 1.3 (3.2-8.9)	8.7 ± 1.3 (5.4-11.6)

### Anatomical relationship between the lumbar plexus and the major psoas muscle

**Figure 2:**
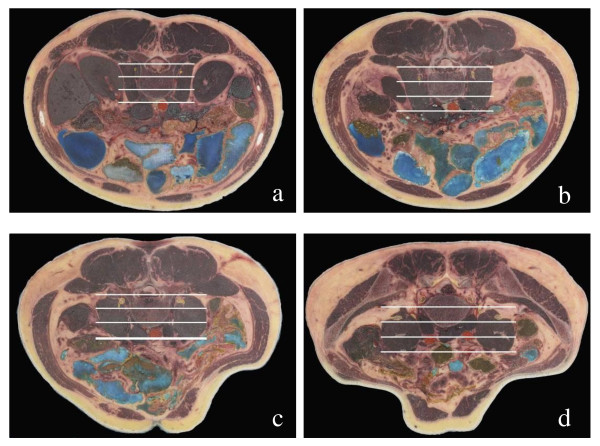
**The relationship between the lumbar plexus and the psoas muscle; show the lumbar plexus always located in the posterior the third of psoas major muscle in all cross section of lumbar the lumbar plexus nerve was marked by yellow circle**. a: Cut image at L2; b: Cut image at L3 displays the L1 and L2 nerve roots; c: Cut image at L4; d: Cut image at L5 displays the L4 and L5 nerve roots;.

During operation, the major psoas muscles should be dissected to expose the vertebral body. According to both the anterior border of the major psoas muscle and the space between the major psoas muscle and the lumbar quadrate muscle, the major psoas muscle could be fractionated into three equal parts that would be helpful in judging the location of the lumbar plexus. From our study, the lumbar plexus was found to always be located in the 1/3 posterior aspect of the major psoas muscle at different sections (Figure 2). Hence, injury can be avoided by incising at the ventral 2/3 of the psoas major.

### 3D reconstruction of the lumbar plexus

**Figure 3:**
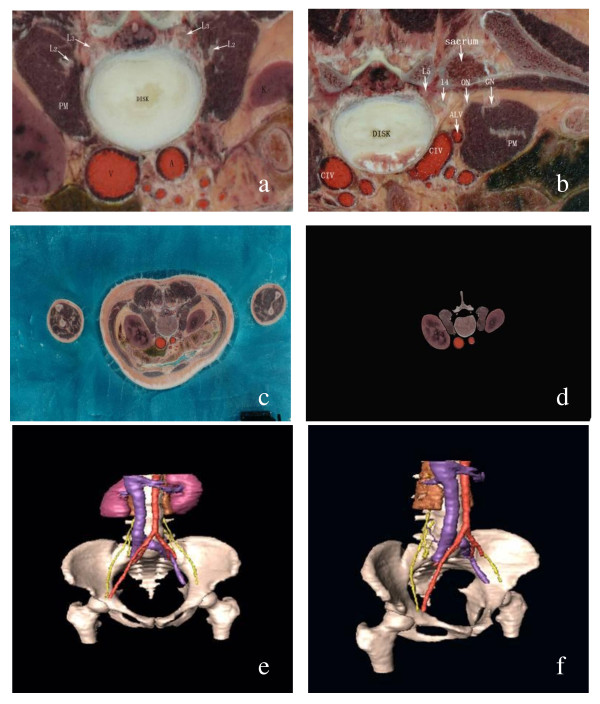
**Three-dimensional reconstruction of the lumbar plexus**. a: Image of a section of L2-L3 vertebra disc in VCH-F 1 dataset; b: Image of a section of L4-L5 vertebra disc in VCH-F 1 dataset; c: original image of VCH-F 1; d: region suction by the Photoshop CS; e: The 3-D reconstruction of the lumbar plexus and its adjacent structures (Anterior view) f: left oblique view L2: L2 nerve root; L3: L3 nerve root; L4: L4 nerve root; L5: L5 nerve root; K: kidney V: inferior vero vein; A: aortic artery; CIV: common iliac vein; ALV: ascending lumbar vein; PM: psoas muscle.

In the Digital-visible database, all sectional images were clear from the level of lumbar vertebrae to the level of the superior segment of the femur, such that the bone tissue, major psoas muscle, kidney, fibrous connective tissue, nerves, and vessels were all discernible. The images of these tissues--nerve roots of L1-L5, the lumbarsacral trunk (LST), the abdominal aorta and its branches, and the inferior vena cava and its branches--could be viewed clearly and consecutively. The static display of the 3D reconstruction images was entirely preserved and then reconstructed regionally. The benefit of the reconstructed images included a clear display of the following: (1) the relationship between the lumbar plexus nerve or its main branches and important blood or vertebral body, (2) the relationship between the lumbar plexus and the major psoas muscle, and (3) the convergence of the lumbar plexus and the lumbarsacral trunk (Figure 3).

## Discussion

The transpsoas approach, which can also be used in the setting of revision arthroplasty or in the conversion to lumbar arthrodesis, involves accessing the anterior spine through the iliopsoas muscle. This way, dissection around the retroperitoneal vessels, as well as in most abdominal structures (e.g., visceral organs, ureters, hypogastric plexus), can be avoided. Risk to the lumbar nerves and plexus is present with this approach. Therefore, careful neurophysiologic monitoring is critical, and comprehensive anatomic data on the lumbar plexus are important [10].

### Location of the lumbar plexus and its clinical meaning

Lumbar plexus nerves were found to be arranged regularly: the nerve roots exit the intervertebral foramina and go down from L1 to L5, the upper nerve roots are always lateral, and the lower nerve roots are opistho-medial. Such anatomical arrangement indicates that with the anterior lumbar approach, injury to the lateral lumbar nerve at the lower level of the lumbar vertebrae would cause symptoms related to lower lumbar plexus injury. This was kept in mind when the injury location of the lumbar plexus was determined. The lumbar nerve goes downward from an angle of 20° at the upper level to 40° at the lower level. According to such an arrangement, dissection of the major psoas muscle should be done at the lower border of the transverse process to avoid injury to the nerve.

### Selection of the landmark for anterior approach to the lumbar vertebrae

The lumbar plexus serves as one of the most important anatomical structures of the anterior approach, and its injury greatly affects patients. Therefore, its location should be considered from the beginning of the approach decision and before the exposure of the lumbar vertebrae. However, the lack of reliable anatomical landmarks at the posterior belly presents problems. This study confirms that the lumbar plexus is closely related to the transverse process. The transverse process, located between the major psoas muscle and the lumbar quadrate muscle, could serve as the landmark for the lumbar plexus during operation. Several important notes were remembered in the course of the study: (1) dissection and exposure of the major psoas muscle should be marked by the lateral border of the transverse process and vertebra pedicle; (2) the self-help retractor should not be placed at the superior articular process and transverse process; (3) the proximal end of the vertebra pedicle should always be approached from the dorsal surface of the lumbar vertebrae; and (4) even though it is easy to approach the lumbar plexus, the lumbar nerve root should be exposed completely to avoid injury to the lumbar plexus at the extreme lateral approach to the intervertebral discs.

### Relationship of the major psoas muscle and the lumbar plexus

After exiting the intervertebral foramen, majority of the lumbar nerves traveled through the major psoas muscle. Some of them formed the plexus within the major psoas muscle. Branches then exit from the anterior surface or lateral border of the muscle. Kirchmair et al. [11] studied the relationship of the lumbar plexus and the psoas muscle and demonstrated that there are two locations of the lumbar plexus that may be encountered in clinical practices: locations within and posterior to the psoas major muscle. However, the latter situation represents a minor variant. At the anterior approach to the lumbar vertebrae, sites that might complicate an injury of the femoral nerve are generally located in the part inferior to the peritoneum, between the peritoneum and the iliac muscle, or between the major psoas muscle and the iliac fascia. Such injuries to the femoral nerve are mainly caused by major psoas muscle hematoma. Sotiris [12] reported two cases of the anterior approach of lumbar vertebral fusion that complicated a tension injury of the femoral nerve. They determined that this was caused by the extended duration of tension of the major psoas muscle during operation, which resulted in insufficient space for the activity of the femoral nerve. A similar situation was observed in this study. This encouraged us to maintain a relaxed major psoas muscle by bending the hip to avoid excessive tension of the major psoas muscle during anterior approach of the lumbar vertebrae. Thus, there was minimal consequence on the femoral nerve. The lumbar plexuses were wrapped by fascia at the deep surface of the major psoas muscle and tightly connected with it. Owing to this, an injury might occur during manipulation of the major psoas muscle. Excision of the extreme-lateral intervertebral discs requires attention to the relationship of the lumbar plexus to the major psoas muscle. If such disc is inferior to L4, lateral retraction of the major psoas muscle along the transverse process by the interspaces of the major psoas muscle and the lumbar quadrate muscle is required. The lumbar plexus is located just posterior to the major psoas muscle, so as much exposure as possible of the lumbar nerve root was required. The lumbar plexus was found to be closely related to the major psoas muscle in the view of body surface positioning and operational mark. This relationship should always be considered during dissection or traction.

### Significance of the anterior approach safety zone

Mono et al. [13] studied the problem of the safety zone and the lumbar plexus via retroperitoneum by laparoscope. The safety zone was concluded to be L2-L3, as well as L4-L5, if injury to the genifemoral nerve is ignored. At L5-S1, the risk of injury was higher if the routine approach was chosen because of the iliac vessels present at the lateral surface. Another challenge was going through the space between the major psoas muscle and the lumbar quadrate muscle and drawing the major psoas muscle forward to reach the lateral to lumbar vertebra. The nerve roots of L4 and L5, the femoral nerve, and the obturator nerve presented and formed a dangerous area for this approach. Thus, sufficient exposure of the lumbar plexus is required. Entering the lumbar vertebrae required pulling away or incising the major psoas muscle, but it was impossible to localize the exact point of the lumbar plexus during operation. Hence, the position of the lumbar plexus versus the major psoas muscle was considered critical. In this study, entering the retroperitoneum was found to reveal the anterior and posterior borders of the major psoas muscle. According to this finding, the position of the lumbar plexus versus the anterior and posterior borders was thought to serve as a landmark to incise the major psoas muscle and safely enter the lumbar vertebrae or intervertebral discs. Choosing the correct place to incise the major psoas muscle is very important. The approach to such a place should avoid injury to the lumbar plexus during the process of dissecting or passing through the major psoas muscle. According to the sectional anatomy on the lumbar plexus and major psoas muscle done in this study, the location of the lumbar plexus at the major psoas muscle was comparatively constant. This made it possible to determine the 2/3 ventral part of the major psoas muscle and to reach the safe area of the intervertebral space. The lumbar plexus was then drawn backwards. It is suggested that 1/3 dorsal parts of the major psoas muscle must be retained to avoid injury to the nerve roots. In 2004, Bergey [14] reported on the anterior approach to the major psoas muscle at the lumbar vertebrae by laparoscope. He emphasized incising the major psoas muscle rather than incising at the medial border of the major psoas muscle then drew it. Thus, injury to the lumbar plexus caused by excessive tension of the major psoas muscle can be avoided. He also recommended retaining the 1/3 dorsal part of the major psoas muscle to avoid damage to the nerve roots. Since at the level of L4-L5 intervertebral discs, nerve roots of L3 pass over the lateral border of the intervertebral discs, injury at the 1/2 ventral part of the major psoas muscle was possible. To minimize the risk of Extreme lateral interbody fusion (XLIF) to the lumbar plexus, the dilators should enter the psoas at the junction of the anterior and middle thirds. A radiolucent blade or tubular retractor system is placed over the largest dilator and docked on the lateral aspect of the disc space [15]. These results were similar to our findings.

At the lateral approach, the location of the L4-L5 nerve is either near the inferior border of the vertebral body or near the inferomedial to the major psoas muscle, and not at the upper lumbar vertebrae. Regardless of dissection of the medial border of the major psoas muscle or dissection through the major psoas muscle, exposing the lumbar plexus to reach the lateral and anterior aspect of the vertebrae above L4 was not necessary. This is as long as surgeons are aware that the lumbar plexus is tightly adhered to vertebral body and that appropriate traction should be retained to avoid injury to the lumbar plexus.

### Significance of 3D reconstruction of the lumbar plexus

Unfortunately, it is difficult to determine the location and adjacent space relation of the lumbar plexus from sectional images of the anatomical layers. If surgeons want to visualize such space location from a two-dimensional image, they would have to speculate carefully and precisely, but this method is problematic. Currently, this method takes advantage of the 3D reconstruction technique to review the anatomical structures of the lumbar plexus from any angle and thus understand the lumbar plexus and its surrounding tissues. The anatomical data presented herein might prove useful for the anterior approach to the lumbar plexus and help avoid injury during surgery.

## Conclusions

The psoas muscle can be considered as a surgical landmark since incision at the ventral 2/3 of the region can prevent lumbar plexus injuries for procedures requiring exposure of the lateral anterior of the lumbar. The transverse process can be considered as a landmark and reference in surgical operations by its relative position to the lumbar plexus. 3D reconstructions of the lumbar plexus based on VCH data provide a virtual morphological basis for anterior lumbar surgery.

## Competing interests

The authors declare that they have no competing interests.

## Authors' contributions

SLu and Z-hD carried out the anatomy of cadaver, participated in the acquisition and analysis of data, or and drafted the manuscript. Y-zZ carried out the 3-D reconstruction of lumbar plexus. Y-qX and SC conceived of the study, and participated in its design and coordination. All authors read and approved the final manuscript.

## Pre-publication history

The pre-publication history for this paper can be accessed here:

http://www.biomedcentral.com/1471-2474/12/76/prepub
